# Disseminated cysticercosis: a case report and review of the literature

**DOI:** 10.1186/1752-1947-2-137

**Published:** 2008-04-30

**Authors:** Ashish Bhalla, Ashwani Sood, Atul Sachdev, Vandna Varma

**Affiliations:** 1 Department of Medicine, Government Medical College & Hospital, Chandigarh, India

## Abstract

**Introduction:**

Cysticercosis is a common tropical disease. One of the uncommon manifestations of cysticercosis is its disseminated form.

**Case presentation:**

We report an immunocompetent patient with disseminated cysticercosis, who had involvement of the brain, subcutaneous tissues, skeletal muscles, right orbit and thyroid gland. In addition, this patient developed a serum sickness which responded to therapy.

**Conclusion:**

Wide spread dissemination is a rare complication of cysticercosis. A planned approach to therapy is required.

## Introduction

*Cysticercus cellulosae *are the larval forms of the tapeworm *Taenia solium*. The adult tapeworms are found in the small intestine of humans, the definitive host, and the larval forms are found in the skeletal muscle of the intermediate host, the pig. To develop cysticercosis, a human has to replace the pig in the *T. solium *life cycle and the eggs must mature within the human small intestine as they would do in the pig's intestine. Entry of the eggs into the human small intestine may occur through autoinfection or by ingestion or inhalation of egg-contaminated food or water. Finally these cysticerci spread through the intestinal wall and are carried by the blood stream to muscles, brain and subcutaneous tissues, leading to clinical manifestations [1].

Disseminated cysticercosis (DCC) is an uncommon manifestation of a common disease. Fewer than 50 cases have been reported worldwide, the majority being from India. In a study of 450 cases of cysticercosis only one case of disseminated disease was seen [2]. This case is remarkable because of the involvement of the thyroid gland and the development of a serum sickness-like illness associated with DCC.

## Case presentation

A 35-year-old woman from Haryana presented with generalized tonic-clonic seizures. She was treated with antiepileptics and became seizure-free. She had also noticed swellings all over her body which had gradually increased in number and size over the previous year, and there was proptosis of her right eyeball. She also had fever and arthralgia. On examination there was symmetrical generalized hypertrophy of the limbs, most prominent in the calf muscles, and also affecting trunk, neck and facial muscles. There was muscle tenderness with increased pain on movement of the joints.

Investigations revealed hemoglobin of 12.5 gm%, total lymphocyte count (TLC) of 12,800 and differential leucocyte count (DLC) of P80%:L20%. The erythrocyte sedimentation rate (ESR) was 40 mm/hour. The level of serum creatinine phosphokinase was 150 (normal value 200). In addition, urine tests showed the presence of proteinuria without any active sediment on microscopy. Routine biochemical investigations revealed normal glucose, renal and liver function tests. The tests for rheumatoid arthritis (RA) factor and antinuclear antibodies were positive but the patient did not have any other symptoms to suggest a diagnosis of rheumatoid arthritis or lupus. Tests for HIV using enzyme-linked immunosorbent assay (ELISA) were negative for both HIV 1 and 2. Electrocardiogram (ECG) examination revealed a right bundle branch block and a right axis deviation. An echocardiogram failed to show cysticerci in the heart. X-rays of the skull and extremities were normal. There was no radiographic evidence of calcification in the muscles. Ultrasound examination of the orbit and neck was performed, revealing multiple swellings in the orbit, thyroid gland and strap muscles of the neck. Fundus examination was normal. Perimetry was also within normal limits. Magnetic resonance imaging (MRI) scan showed multiple cysts in the brain, scalp tissue, orbit and neck muscles. There was no evidence of hydrocephalus. Biopsy of a subcutaneous swelling was taken from the right forearm. Cysts poured out as soon as the skin was incised. Histopathological examination confirmed that the cysts were of *C. cellulosae*.

The patient was treated with prednisolone 1 mg/kg of body weight 1 week prior to the initiation of albendazole therapy instituted at a dose of 15 mg/kg. The patient was observed for 5 days prior to discharge. The symptoms improved and albendazole was continued for a total duration of 30 days. There was objective evidence of improvement with reduction in the size of the swellings. There was no deterioration in neurological or intellectual status and no appearance of new crops of cysticerci. To our surprise her fever and arthralgia disappeared without the use of anti-inflammatory agents. This improvement lasted for 6 months, at which time there was an increase in the size of existing swellings plus development of new crops of swelling. The fever and arthralgia reappeared. The patient was again primed with steroids and given praziquantel therapy. The patient responded and was discharged after 10 days of observation in hospital. Antiepileptic treatment was continued.

On followup after 1 year, no new swellings or any apparent increase in the size of the residual swellings were reported, there was no further fever or arthralgia and the patient remained seizure-free.

## Discussion

Widespread dissemination of cysticerci throughout the human body was reported as early as 1912 by British Army medical officers stationed in India [3]. Priest, in 1926, described probably the first case of extensive somatic dissemination of *C. cellulosae *in a British soldier who had swelling of his muscles, epileptic seizures, mental dullness and widespread subcutaneous nodules [4]. Subsequent studies failed to highlight this form of clinical presentation, because of its relative rarity [4]. An extensive search of the the English literature on PubMed has yielded 22 cases reported by Wadia et al. [4] and an additional 16 cases reported up until 2006.

Human cysticercosis is caused by the dissemination of embryos of *T. solium *from the intestine via the hepatoportal system to the tissues and organs of the body. The organs most commonly affected are subcutaneous tissues, skeletal muscles, the lungs, the brain, eyes, the liver and occasionally the heart. Widespread dissemination of the cysticerci can result in the involvement of almost any organ of the body.

The main features of DCC include intractable epilepsy, dementia, enlargement of muscles, subcutaneous and lingual nodules and a relative absence of focal neurological signs or obviously raised intracranial pressure, at least until late in the disease [1,3]. Absence of calcification in soft tissues and the head on radiological examination and the presence of living cysticerci at biopsy or autopsy are important findings, although the latter has not been sufficiently observed. Pseudohypertrophy of the muscles is the most common presentation of DCC, followed by palpable nodules and seizures [4]. Our patient presented with epilepsy, subcutaneous nodules and nodules in the thyroid gland and did not have pseudohypertrophy.

Computed tomography (CT) scans and MRI are useful in anatomical localization of the cysts and in documentation of the natural history. MRI is more sensitive than CT as it identifies scolex and live cysts in cisternal spaces and ventricles and identifies the response to treatment [5,6]. Unenhanced CT scans of muscles can show innumerable cysts standing out clearly against the background of the muscle mass in which they are embedded, the CT image appearing like a honeycomb or leopard spots [5]. In our patient the CT scan had a characteristic 'starry sky' appearance but did not reveal any calcified foci in muscles.

In addition to having subcutaneous nodules our patient also had cysticerci in the thyroid gland, as evidenced by ultrasound examination of the neck. Involvement of the thyroid gland has not previously been described in the literature. We could not demonstrate definitive histopathology, as biopsy from the thyroid gland could not be performed.

In addition, the symptoms of fever and arthralgia have not been previously described and are unique to our case. Our patient had fever, arthralgia and proteinuria, and was positive for non-specific RA factor and antinuclear antibodies, suggesting a type III hypersensitivity reaction which has not been reported in other cases.

Management of DCC includes symptomatic treatment of central nervous system lesions using steroids and antiepileptics. In patients with raised intracranial tension, surgical removal of cysts and ventriculoperitoneal shunting can alleviate symptoms.

Pharmacological management with the cysticidal drugs praziquantel and albendazole is indicated as they help by reducing the parasite burden [7]. These drugs hasten the death of the cysts, which may occur even in the absence of such treatment. Pharmacological treatment may be associated with severe reactions, which may result from enlargement of cysts, massive release of antigens causing local tissue swelling and generalized anaphylactic reaction [1]. Priming with corticosteroids before starting the cysticidal drug [1,4] decreases the incidence of such complications.

## Conclusion

It is important to recognize DCC clinically and to perform appropriate radiological investigations, as this condition needs planned therapy. Patients who are on treatment and who have active cysts remain at risk of serious complications.

## Abbreviations

CT: computed tomography; DCC: disseminated cysticercosis; DLC: differential leucocyte count; ECG: electrocardiogram; ELISA: enzyme-linked immunosorbent assay; ESR: erythrocyte sedimentation rate; MRI: magnetic resonance imaging; RA: rheumatoid arthritis; TLC: total lymphocyte count.

## Competing interests

The authors declare that they have no competing interests.

## Authors' contributions

AB compiled the data and prepared the manuscript. ASo oversaw clinical management of the case. ASa was in overall charge as Head of Department. VV undertook all radiology. All authors read, revised and approved the final manuscript.

## Consent

Written informed consent was obtained from the patient for publication of this case report and any accompanying images. A copy of the written consent is available for review by the Editor-in-Chief of this journal.
